# Current Challenges for Healthcare Services and the Opportunities Created by the Marketing Abilities

**Published:** 2008-02-25

**Authors:** F Popa, T Purcărea, LV Purcărea, M Raţiu

**Affiliations:** * ‘Carol Davila’ University of Medicine and Pharmacy, Bucharest Romania; ** American University, BucharestRomania

## Abstract

Life is changing dramatically, market position as part of life is becoming more and more important, and marketing, considered a key cultural architect of nowadays that involves voluntary relation exchanges between the communicating partners, is placing the patient in the center of most adequate action towards the medical future which represents his life quality. We think that the moment has come to resort to marketing as a new method to identify innovation opportunities in healthcare services' delivery, considering that the high quality of healthcare services, answering to demands of healthcare systems' consumers, represents a well recognized priority for the European citizens. Consequently, the model of ‘healthcare business’ has to rely on the value for patient by creating competition concerning the results at medical condition level.

## Adequate Patient's Experience Management

People acting in marketing–important cultural architect of nowadays [1]– intend to offer a variety of services to attract new category of clients, differentiated in segments, and to create adequate marketing mixes to each target market. In order to become reliable builders of healthcare services, marketing managers within this sector, have to find adequate answers to the following questions:

- Do we understand that in the centre off all marketing operations, specific to our organizations, there must be the patient?
- How consistent is the ‘brand’ message of our organization from the point of view of underlining the focus on patient?
- Is the service for the patient really in the centre of our marketing strategy?
- Do we really offer the solutions that the patient is expecting for?
- How well do we master the contact points with the patient and persuade him to accept our proposed solution?
- Do we succeed to see beyond his service acquisition behaviour?
- Do we have the capacity to determine the patient ‘to journey in the medical European future’,together with us?

Focusing on the patient is realized using relevant tools and techniques, acknowledging the strategic relation that he desires. Adequate effort's focus is only possible with the help of a specific set of capabilities which enable the knowledge and decoding of patient behaviour, as the human resources involved in healthcare services delivery are responsible for the engagement made in the name of the performing brand entity. It is well known that patient’s experience will endure in absence of a connection between those who make the engagement and those who must comply with it.

In this respect, it is worth to mention Henry Ford's words who stated that ‘*a business, totally dedicated to serve the clients, will be confronted to one single problem regarding their profit: it will be ‘embarrassing’ large’*. 

A Deloitte study – regarding the service revolution and its impact on the organizations profitability and about the global producing industries, in general – shows that, a lack in capabilities of planning, management and monitoring more efficient the services activities, the customer relations, may stop or reduce the performance for many companies [2].

## Strategic Proactive Intercession and Orientation towards the Patient

It must be kept in mind that ‘without the external deposit’ (the need of health, the market, the patients – natural resources of the organization) nothing would be possible from strategic point of view, and the study about it, as far as it proves to be a positive one, enables the making of a ‘product – healthcare service’, as the pair ‘product – service’, respective ‘production – productivity’, represents the marketing, technical and organizational instruments acquired from the human, informational and equipment resources of the healthcare organization [3].

The healthcare organization must define its ‘competition battlefield’, by identifying and studying its strategic segments. In this respect it is preferable the new proactive intercession, which assumes that the instrument used to satisfy a health need is conceived by the patients by means of iteration, and the quested instrument is stopping the ‘image–reaction–change’ process. The organization can open in this way, through fetched innovation, its own market and to disturb the competition game in its own advantage, with a new substitution force. The transpose to practice of this intercession for the healthcare organization competitiveness, implies to comply with some operational considerations.

The most secure way to competitiveness is the one that associates in a harmonic manner the productivity (the capacity to produce more with fewer tools) and the effectiveness (the capacity to better respond to environment expectations, especially to the ones of the clients) [4].

For example, the above mentioned Deloitte study, shows that, in the healthcare services sector (and in the medical equipment industry), the clients become more and more pretentious, raising the level of exigency and pretending excellence in service delivery [5]. In this respect, the same study– regarding to services revolution and its impact about the profitability of organizations and about global production industries in general – emphasizes that, customer requirements to deliver the service in the same day (instead of the next day or later) will become more and more frequent, combined with agreements about the quality of delivered service, witch will raise risks–as well as rewards – for the companies within this domain. For certain companies, such as *Siemens Medical Solutions* – a leader in the delivery of imagistic systems, therapeutically equipments, auditory and molecular diagnosis instruments – these requirements usually imply to create a distribution system and *a more complex and expensive service network, in order to ensure a better closeness to the client and a faster response*. The improvements required in the processes and systems of management and optimization of these networks will represent a challenge even for the market leaders in this domain, in the next years.

## Competition Requirements in Terms of Value Offered to the Healthcare Consumer

Healthcare service providers are acting in fact, in a business of delivering services to patients. It is very important to understand this aspect that guides the organizational thinking about: who is the client, what does he needs, how is the value for client created (the alignment in organization vision about the value and the actual value is a precondition for excellent performance), how everything should be organized.

It is said that ‘a doctor who does not have information about the medical past of the ill person and a patient who can not furnish it, are in danger to prescribe, respectively to receive an inadequate treatment’ (unknown author). Even in the developed countries, recent authorized signals emphasize the chronic deficiencies of the healthcare system: increasing costs, reduced client satisfaction, raising deficiencies regarding quality, limited coverage [6].

It is considered that today, the real challenge for all those involved in the services industry (including also the healthcare services), no matter the position, is how to respond when they are contacted, being essential the provider's training as an marketer, who knows well the five keys of customer driven marketing (‘5 Keys to Customer–Driven Marketing, CRM tells you what customers bought. But to meet their needs, you have to know why’, DMNEWS, 22.11.2006), respectively: ask the customers what they really think; focus on what they think it's important; make sure you can act based on customer feedback; get feedback on a permanent basis; create feedback loops in your entire company. It should be noticed that the year 2007 has been considered, from the services approach point of view, as a ‘year of differentiation’ [7], starting from the recommendation that ‘if you cannot make the service different, than differentiate yourself by offering more services’.

In the evaluation of healthcare services, the patients are using some criteria, which represent in fact defining elements of healthcare services quality: accessibility, specificity, continuity, efficiency, effectiveness of healthcare services; patient orientation; safety of healthcare environment; and daily schedule. For the patients, the quality and the correctness are inseparable; a medical unit having a doubtful correctness is an organization which is performing poor quality medical services. Seriousness in carrying out medical services grants for the medical organization credibility.

Medical organizations interested to reach important performances in terms of value for the healthcare consumers, must ground their activity on the following elements [8]:

- Working out high performance standards regarding the quality of their own medical services delivery;
- Supervision of healthcare services delivery process, using specific methods: mystery patient test, complaints and suggestions analysis, patient opinion research, auditing teams of healthcare service and so on;
- Constant involvement of top management in quality matters by monthly analysis not only of financial results, but also on service delivery level;
- Creating necessary conditions to support and reward medical personnel, by focusing on patient satisfaction, and employees, too; as the relations with personnel reflect the relationships with patients.

For instance, ‘Victor Babes’ Diagnose and Treatment Centre is the first private medical centre in Bucharest which has implemented a quality management system according to European standard SR EN ISO 9001:2000. For diagnosis, supervision and healthcare, the centre has primary and specialist doctors who are part of a high competent and professional team, very well trained in theory and practice; and this represents a real high quality service guarantee [9].

This competition for offering value to healthcare service consumer requires for the healthcare service providers to follow a number of strategic and organizational imperatives (redesigning the business based on medical conditions; selecting the medical product type and mix; surrounding organizations of integrated medical practice units; creating a distinct strategy in each practice unit; measuring the results, the experience, the methods and patients' attributes per practice unit; resorting to single bills and new approaches in fixing prices; providing market services based on excellence, uniqueness and results; local and geographical growth, in key areas.

## Value for the Consumer, as the last Judge for Adequate Definition of the Medical Condition 

Martin H. Fischer, psychology specialist once said: ‘the patient is not interested in the physician's scientific knowledge; he only wants to know if the physician is capable to cure him’. In healthcare services delivery, the value for the patient can be understood only at medical conditions level, because: it is preponderant depending on the service delivery manner in the given circumstances; it is resulting out of the complete set of involved activities and specializations (in doesn't matter the individual roles, abilities or functions, but the general result); it is determined for each aspect of the healthcare by the harmonization of necessary abilities and functions.

For example, in surgery, the value depends not only on the surgeon, but also on the anesthetist, medical assistant, radiologist, technicians and so on, all of them performing adequately; it matters how skilled is the chirurgical team, but it is crucial the general healthcare cycle; the patient results will suffer if: his problem is not accurately diagnosed; he is not adequately prepared; his recovery and rehabilitation are not well managed. The impact of healthcare cycle could be more important without chirurgical intervention or when the case is treated differently, and even more important if preventive healthcare and consultancy are provided over the time so that treatment is no longer necessary.

As a consequence, in offering medical services with high value for the patient, the satisfaction of healthcare organization employees has a crucial role. The providing medical personnel is responsible for offering quality healthcare services and for delivering satisfaction to the patient. Thus, it becomes more obvious that the personnel's attitude and behaviour may increase or decrease the value for the patient and the reputation of the organization that are providing healthcare services. Or, in other words, the healthcare organization success depends on how the managers are leading with competence, creativity and imagination their employees [10].

Consequently, we can consider that, the relevant business in delivering healthcare services is the medical condition looked upon the complete healthcare cycle. It mustn't be forgotten that the last judge in the adequate defining of medical condition is the value for the patient (every provider has to define afterwards, clearly and explicitly, the medical condition set that he participates in, any of these conditions being placed in the healthcare cycle; next it is to conceive a strategy, to organize the healthcare delivery and to evaluate the results).

For example, by continuously improving the value of healthcare services delivered to the patients and starting from the idea that the most devastating misfortunes pounced upon us during lifetime are actually produced by us, the Centre of Diagnose and Treatment ‘Dr. Victor Babes’ has initiated the following message to its patients: ‘Starting from today, we want to stop all your pains’, ‘Your health is our goal’. It is offered the opportunity to monitor and to ameliorate patient's health and, through this, to ensure the quality of his own life and of his beloved ones.

It can be considered that the essential problem in business is the focus on products and services that create unique value, which implies, in hospitals and other healthcare providers' cases, a significant change regarding the way of thinking about treating the patient who enters their door.

In healthcare services, as Porter mentioned, the need for services strategic choice has been avoided because of the lack of information and the lack of results registration. Strategic focus doesn't mean a narrow specialization, but to pursue excellence and profound penetration of chosen domains. Also, significant debates [11] regarding marketing developments have outlined the statement that ‘the real marketing future is in accountancy ’ (Alexander Black, from Chicago Business Consulting Group Inc., with the occasion of Conference ‘Marketing in 2010: Growth Strategies for the Future’, in his presentation: ‘Accountability in 2010: Taking Marketing ROI to the Next Level’), the goal being not the maximization of ROI (its measuring in marketing terms should be scalable, residual, and interdependent), but the profit maximization by increasing long term value for the customer.

## Marketing and Innovation in the Physician – Patient Co–production (Healthcare Services Sector)

‘The new economy’, that we are assisting to nowadays which affects all sectors including healthcare services domain is imposing new competition rules, new organization methods, new commitments about the relations and communication extension, new challenges for the marketing management. In the knowledge economy, cleverness and dynamism, innovation and technology are considered to be essential instruments [12].

These challenges created by ‘the new economy’ are affecting all marketing areas–including medical services marketing –which must align to these changes and must use the essential instruments (cleverness, dynamism, innovation and technology), without that, the failure is sure [13].

For example, the best innovators know how to spend their money efficiently. They use a phased idea-management process that works like a funnel [14]. Thus, Randal Moss, member of Futuring and Innovation Centre (FIC) part of American Cancer Society – ACS, once said: ‘Innovation is not just a process of evaluating and implementing ideas, but a way of viewing challenges, resources, and possibilities that must be infused in the culture of any successful organization. It is based on a continuous innovation process’. In 1997, the society defined a series of very ambitious targets for the year 2015, called ‘goals 2015’, and these become the organization slogan and the weight centre of its innovating efforts.

Taking into consideration the crucial role of innovation within the framework of medical organizations management, we can consider that, *innovation in healthcare services must offer to patients' health, confidence, being perceived as unique*, and must find enough patients willing to pay the costs, producing a plus of economic ideas, and of course profitability. It is not easy to achieve this performance of innovation: it is necessary to identify the goals, to clearly define the target customers and therefore, it is required a marketing strategy that considers the existence of innovating organizational culture, on the background of permanent innovation process improvement.

## Healthcare Services Going Out of the Functional Model: Birth of the Competition in Terms of the Results at Medical Condition Level

Being correct placed within the healthcare services, competition can reduce the costs, improve medical performance and produce superior results for the patient [15]. In this moment, in the developed countries, confronted with the reality that healthcare system is not well aligned to create value for the patient, competition at medical condition level has been started. They consider that the government can do more by changing and developing the key policies, particularly in the information area, and by eliminating restrictive and useless impediments for the competition.

Professional debates that took place in the autumn of 2006, at OECD level [16],revealed some significant aspects, such as:

- *Competition role in hospital services offer*. Market mechanisms can contribute to cost reduction of hospital services offer (limited funds allocated to healthcare services can have a bigger impact), the introduction of these mechanisms is completely compatible with the extensive and equitable access to healthcare services;
- *Preliminary conditions necessary to organize the market mechanisms*: the financial support a hospital benefits must be correlated with the number of patients and with the proposed treatments (the hospitals must be stimulated to treat more patients); selective contract should be authorized (the clients of hospital services must not be obliged to buy services from all hospitals; hospitals that offer services at a higher level should be allowed to receive more patients); other possible providers should be available and must have the capacity to absorb the growing volumes of patients (by eliminating monopoly power); sufficient information have to be gathered in order to judge precisely the services offered by the hospitals (including healthcare quality indicators);
- *Hospital services heterogeneity*. Certain hospital services benefit by competition more than others and this competition comes not necessary from other hospitals. Healthcare services, including hospital services are combining unusual characteristics which could imply excessive expenses if a totally free market approach would be adopted.
- *Different effect of competition for rural hospitals and for extremely complex services*.Hospitals are not all capable to equally benefit from the market forces;
- *Anti–competition restriction in utilization of working resources*. More flexibility in some tasks could significantly improve personnel's productivity;
- *Mobility and consumers' selection*. When the waiting lists are long, it is recommendable to adopt the principle ‘money succeed the patient’ (it contributes to reduce the waiting lists and to increase the production);
- *Competition policy applied to hospitals*. Introduction of market mechanisms implies that public authorities will give attention to market structure (by fusions control) and to providers coordination (by measures against anti–competition agreements).

Most of the time, the healthcare service with the best quality is the service with the lowest price, and the explanation is that when the patient remains healthy or is getting healthy quicker the costs are reduced (by determining the correct diagnosis is avoided an unnecessary treatment and the costs are reduced; by avoiding mistakes or by using excellent surgery that allows the patient to get out of the hospital sooner). As a conclusion, the way in which costs are reduced in healthcare service area, need to allow the increase of service quality. Porter is argues that the only way that works in this respect is the measurement of results (beyond the focus on healthcare methods or processes), that implies:

- Creating the competition regarding the results at medical condition level;
- Escaping the fear feeling that information could be used in mal praxis process – surpassing the phase which presumes that a negative result means that the practitioner hasn't done his job well;
- Understanding the fact that complicated adjustments could be necessary in order to reduce the patient's risk;
- Improvement of results evaluation process to the patients' benefit.

The above arguments are exposed on the background of the increased concern about the identification of new methods and models to reduce the waste and to improve the efficiency in healthcare services sector (‘Accountable Marketing is the new rulebook for that new world’ [17]).

The movement for quality in healthcare systems is considered today a correct step in the right direction, if it is focusing on results. Patients' migration towards excellent providers in terms of medical condition represents a powerful stimulus, which feeds the virtuous cycle of value improvement, in volume and experience.

Healthcare plans that integrate all individual healthcare needs are imperatively necessary as long as competition exists, both at providers' level and at medical condition level. Healthcare plans have an important role in medical registration stimulation, as they will bring more value in the system; they can create stimulus and standards for quick dissemination of medical electronic records for all the actors in the system.

In order to complete satisfy healthcare services consumers' needs, it is necessary to acquire marketing abilities and to understand the patients, to identify their wishes and needs, and to build the confidence which will generate the acceptance of solutions proposed through an European vision (aiming the improvement of citizens health security, the engendering and dissemination of knowledge about health, the promotion of health to improve prosperity and solidarity), in the effort to offer: an efficient answer to health threats, help to prevent diseases, an increased co–operation between healthcare systems to adapt to key aspects about healthcare, and to those aspects which could arise unexpectedly and require urgent attention.

## Conclusions

Changes inside the medical world require indeed a sense of direction, acting at the right time, and now – in the context where the high quality of healthcare services, responding to customers' needs, represent a recognized priority for the European citizens–the time has come to resort to marketing as a new method to identify innovation opportunities in healthcare services delivery. In fact, we are right in the middle of implementing the new European Health Strategy process, which aims at secure, high quality and efficient healthcare services.

Consequently, this is the general context that motivates our proposal to resort to marketing as a new method to identify the innovation opportunities in healthcare services delivery, asserting that the professionals acting in healthcare services area and the patients should work together in order to discover how to satisfy better the latter ones: by understanding them and knowing them very well, thus becoming trustful builders of the healthcare service delivery.

We are totally convinced that the auditors will understand our pleading for a ‘brand’ message that emphasizes the orientation towards the patient, together with the other medical practitioners in the ‘European medical future journey’ to ensure the best experience by adopting adequate marketing strategies and tactics. As a matter of fact, this is our ‘diagnose’, only practice is the one capable to confirm it.
